# 733. Letermovir treatment for refractory or resistant cytomegalovirus infection or disease with concurrent organ dysfunction: an interim analysis of a Phase 2 open label study.

**DOI:** 10.1093/ofid/ofac492.024

**Published:** 2022-12-15

**Authors:** Isabel H Gonzalez-Bocco, Matthew Cheng, Muneerah M Aleissa, Esther I Arbona, Kaiwen Chen, Eric Zhou, Katherine Beluch, Alyssa Cho, Sandra Burchett, Sarah P Hammond, Nicolas C Issa, Amy C Sherman, Francisco M Marty

**Affiliations:** Brigham and Women's Hospital, Boston, Massachusetts; McGill University Health Centre, Montreal, Quebec, Canada; Brigham and Women's Hospital, Boston, Massachusetts; BWH, Boston, Massachusetts; Robert Wood Johnson Medical School, Piscataway, New Jersey; BWH, Boston, Massachusetts; Brigham and Women's Hospital, Boston, Massachusetts; Brigham and Women's Hospital, Boston, Massachusetts; Boston Childrens Hospital/Harvard Medical School, Boston, Massachusetts; Massachusetts General Hospital, Boston, Massachusetts; Brigham & Women's Hospital, Boston, Massachusetts; Brigham and Women's Hospital, Boston, Massachusetts; Brigham and Women's Hospital, Boston, Massachusetts

## Abstract

**Background:**

Cytomegalovirus (CMV) disease is associated with increased morbidity and mortality following solid organ and hematopoietic cell transplantation. Currently, standard of care CMV treatments often have significant myelosuppressive or renal toxicities. Given letermovir’s favorable safety profile when used prophylactically, it may be a safe and efficacious alternative agent for CMV treatment.

**Methods:**

A proof-of-concept, open-label trial was conducted in patients who lacked effective therapeutic options or presented with baseline organ dysfunction. Participants were eligible for enrollment if they were ≥12 years old and had documented CMV infection refractory to treatment defined as failure to achieve >1 log reduction in CMV viral load (VL) (when VL > 500 IU/mL) or lack of clinical improvement for CMV end-organ disease after ≥14 days of standard CMV treatment. Alternatively, patients with severe myelosuppression and renal dysfunction at baseline or genotypic antiviral resistance were also eligible. Participants were excluded if their current CMV infection developed while receiving letermovir for CMV prophylaxis. Letermovir was administered daily (480 mg PO/IV) for up to 12 weeks, with optional additional 12 weeks of treatment for secondary prophylaxis if clinically indicated.

**Results:**

Ten patients met eligibility criteria and were enrolled. Reasons for enrollment included ganciclovir resistance (1/10), refractory CMV infection (6/10), renal dysfunction (7/10), and myelosuppression (7/10). The median baseline CMV VL was 1272 IU/ml [interquartile range (IQR); 925, 2546]. Six patients completed the study, three died due to complications of primary disease, and one discontinued due to diarrhea. Five patients (50%) had documented CMV viremia clearance, with a median time to first unquantifiable/undetectable CMV VL of 13 days [IQR; 9,18] and a median treatment duration of 53 days [IQR; 15,84]. Infections and GI disorders were the most common adverse events (AE), none considered related to study drug. No unexpected AE were observed during letermovir treatment.

**Conclusion:**

Letermovir may be a safe and tolerable alternative for patients with treatment refractory CMV infection or for patients with severe baseline myelosuppression and renal dysfunction.

**Disclosures:**

**Matthew Cheng, MD**, AstraZeneca: Honoraria|Cidara Therapeutics: Grant/Research Support|Scynexis Inc.: Grant/Research Support **Sandra Burchett, MD, MSc**, merck: Grant/Research Support **Sarah P. Hammond, MD**, F2G: Advisor/Consultant|F2G: Grant/Research Support|GSK: Grant/Research Support|Merck: Grant/Research Support|pfizer: Advisor/Consultant|Scynexis: Grant/Research Support **Nicolas C. Issa, MD**, AiCuris: Grant/Research Support|Merck: Grant/Research Support **Francisco M. Marty, MD, SM**, AlloVir: Advisor/Consultant|Amplyx: Advisor/Consultant|Amplyx: Grant/Research Support|Ansun: Grant/Research Support|Avir: Advisor/Consultant|Chimerix: Grant/Research Support|Cidara: Grant/Research Support|F2G: Advisor/Consultant|F2G: Grant/Research Support|Gilead: Grant/Research Support|Janssen: Advisor/Consultant|Kyorin: Advisor/Consultant|Merck: Advisor/Consultant|Merck: Grant/Research Support|Regeneron: Advisor/Consultant|Regeneron: Grant/Research Support|ReViral: Advisor/Consultant|Scynexis: Grant/Research Support|Symbio: Advisor/Consultant|Takeda: Grant/Research Support|United Medical: Advisor/Consultant|WHISCO: Grant/Research Support.

